# Non-Markovian Population Dynamics: Does it Help to Optimize the Chemotherapeutic Strategy?

**Published:** 2010-03

**Authors:** Dmitry A. Kuznetsov, Sergey A. Roumiantsev, Majid Fallahi, Nima Amirshahi, Andrey V. Makarov, Karina S. Kardashova

**Affiliations:** 1 *N.N.Semenov Institute for Chemical Physics, Russian Academy of Sciences, Kosygin St.4, Moscow, Russia;*; 2 *Department of Medicinal Nanobiotechnologies, N.I.Pirogov Russian State Medical University, Ostrovityanov St.1, Moscow, Russia;*; 3 *Department of Molecular and Experimental Hemamatology, Oncology and Immunology, Federal Research Center of Pediatric Hematology, Oncology and Immunology, Leninsky prospect 117, Moscow, Russia;*; 4 *Department of Clinical Hematology, N.I.Pirogov Russian State Medical University, Ostrovityanov St.1, Moscow, Russia;*; 5 *Department of Fundamental and Applied Neurobiology, V.P.Serbsky Institute for Social and Forensic Psychiatry, Kropotkinsky Pereulok, 23, Moscow, Russia*

**Keywords:** chemotherapeutic strategy, population dynamics, drug-tumor selectivity

## Abstract

A non-Markovian theory of population dynamics is to simulate the anti-cancer drug distribution between malignant and the hosting normal cell pools. The model takes into account both the cell life span and the proliferation rate differences. This new simulation approach looks promising for its potential to optimize a chemotherapeutic strategy by choosing the scheme with a higher degree of the drug-tumor selectivity. The pre-test designed simulation mode fits nicely the experimental data on Porphylleren-MC16 (PMC16) pharmacokinetics patterns including the allometric plots revealed for this novel medicinal nanoparticle possessing some anti-cancer potential and intervening into the oxygen-independent ATP production mechanisms.

## INTRODUCTION

### General Background

Drug toxicity to normal tissues and the emergence of drug-resistance are the major factors in limiting responsiveness to chemotherapy of cancer and many parasitic diseases (1–3). A non-Markovian theory of population dynamics in harshly varying object-surrounding environments could be applied to chemotherapeutic systems (4, 5). The original model presented suggests a new policy for improving responsiveness to the phase-specific drugs taking into account their non-discriminative distribution within a cell pool consisting of slow and fast regenerating (proliferating) populations.

### A Non-Markovian Population Dynamics Model to Propose

The dynamics under various drug regimens of populations that differ in life-cycle parameters is simulated using a computer model whose simplest form is given in eq. (a):

(a) x(t)=λx(t−τ)[1−D(t)]

where *x(t)* is the population density at time *t*, *λ* is the birth rate, *τ* is the generation time, and *D(t)* denotes the environmental process, so that *D(t)>0* corresponds to the occurrence of effective concentration of the drug in the system. Using this model the elimination time of malignant population (*T_m_*) and that of the limiting host population (*T_h_*) were estimated, and the elimination coefficient, *Z*, measuring the treatment efficacy, was calculated according to eq. (b):

(b) Z = 1 –Tm/ Th

**Figure 1:**
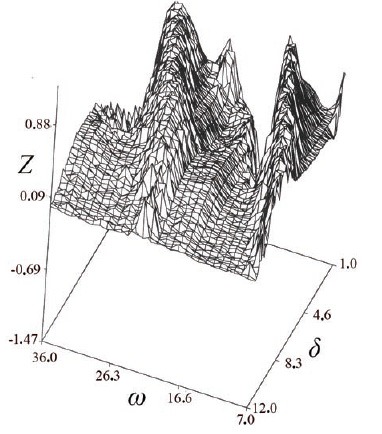
Simulation results of the model for two populations that vary in their life cycle parameters. The elimination coefficient, *Z*, for a malignant and a host population is calculated under different drug regimens represented by the effective period of the drug, *δ*, and by the duration of the drug-free interval, *ω*. Generation time of the malignant cells is 18 hr and their resistant life phase is normally distributed with average 6 hr and variance, *σ = 1*; generation-time of the host cells is 16 hr and their resistant life phase is normally distributed with average 10 hr and variance, *σ = 0.5*; *δ* is normally distributed with variance, *σ = δ/10*, and *ω* is constant; intrinsic birth rate is, *λ = 2*; initial population size is *x(0) = 5*.

Simulation results (Fig. 1) show that treatment efficacy is a nonmonotonic function of the relation between the cell generation time and the period of drug administration, with maximal occurring when the limiting host cell cycle length is a multiple of the chemotherapeutic period. Analytical results further show that in fully periodic systems elimination time, *T*, is given by eq. (c), for *τ>δ>τ/2*:

(c) T = τω / |τ−(δ+ω)|

In eq. (c), *δ* is the duration of the period in which the drug is effective, and *ω* is the period in which the drug dosage is below efficiency. The point, *τ = δ+ω*, is a singular point with *T* being infinite.

To proceed the simulation data, the most common drug distribution and cell proliferation patterns (4, 5) were treated using a Sigma Plot CX600 algorithm with the LabRun-06 software processed in the HP 9107-2SQ Graph Design Analytical Unit.

**Figure 2:**
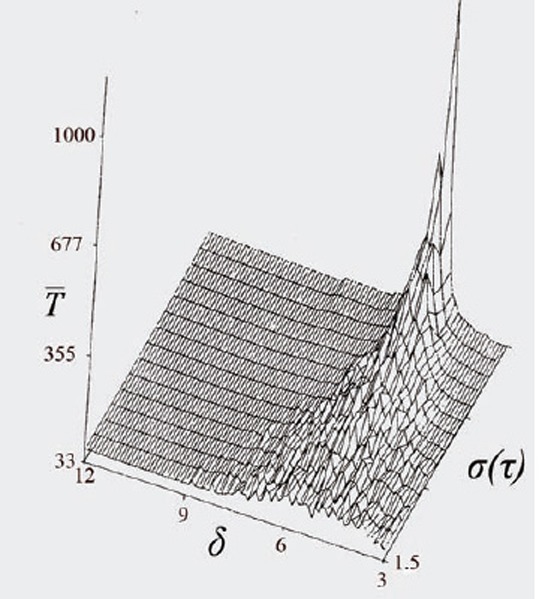
Simulation results of the model in which the average elimination time, *T*, is plotted as a function of variance in the generation time, *τ*, for regimens that differ in drug’s effective period, *δ*. Cell-cycle length is 12 hr; the duration of the drug-free interval is 7.5 hr; other parameters as in Figure 1.

These and previous results point out the harm in maintaining effective phase-specific drug dosage for relatively long periods: Figure 1 shows that increasing duration of individual drug-pulses damps down the synchronization effect, so that the ability to discriminate between the malignant and the host populations diminishes greatly. Further simulations (Fig. 2) suggest that elimination time decreases with increasing variance in cell-cycle length. Thus, due to larger variance in their generation-time (3, 6, 7), neoplastic populations are expected to be eliminated faster than the normal limiting populations, when the frequency of drug administration is a multiple of the average generation-time of the latter; this will be so even if the two populations have similar average generation time. The above results suggest that cytotoxicity to normal tissues can be reduced by a policy of short drug pulses whose period is a multiple of the limiting host population's generation time.

That increasing variance in the temporal parameters reduces the average extinction time may bear upon chemotherapeutic strategies of noncytotoxic drugs. Figure 2 implies that *regimen resistance* can emerge, under fully periodic drug regimens, by selection in the pathogen’s population of lines whose generation-time is similar to the period of drug application; synchronization with the chemotherapeutic period offers these lines a temporal refuge from the detrimental effect of the drug. Regimen resistance can be limited if artificial stochasticity is introduced in the drug regimen by randomizing the timing of drug administration.

So the strategic decision making algorithm described is applicable to the numerous stochastic drug-cell/tissue distribution models (5, 6).

Once the drug-receptor specific recognition involved (1, 2), this algorithm might be complicated in a way of the Penmann-Dalbreaux non-parametric / multi-variant discriminative approximation developments [5]. In its present form, nonetheless, the model proposed is surely suitable for all known cases where the distribution of drug receptors between malignant and the tumor-surrounding intact cells is about a non-discriminatory equal.

This work is a first report to trace a direct link between

(a) The malignant/normal cells cytostatic drug distribution patterns, i.e. tumor-drug targeting selectivity, and

(b) The prediction-making power of a non-Markovian population dynamics in its invariant version.

This allowed to propose a simple and efficient computational simulation method (Figures 1, 2) suitable for the in-advance prediction making optimization of chemotherapy schemes in a vast variety of cases as long as the drug receptor(s) distribution doesn’t show a marked asymmetry in a mixed pool of differentiated and malignant cells. An obvious advantage of the model proposed is that it takes into account an easily available data on the cell life span values and proliferation rates.

**Figure 3:**
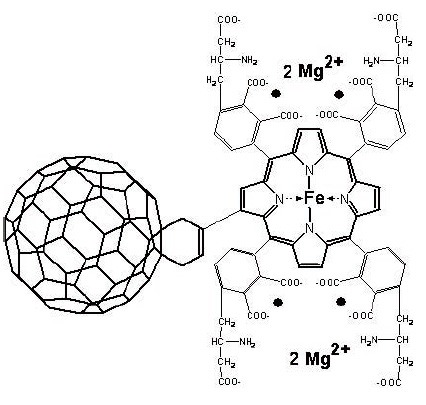
Porphylleren – MC16 (PMC16) (Buckminsterfullerene (C_60_) – 2 – (butadiene – 1 – yl) – tetra (0 – γ – aminobutyryl – o – phthalyl)-4-cyclohexyl-2-ferroporphyrin).

Porphylleren – MC16 or, in brief, PMC16 (Figure 3), a recently developed pharmacophore based on the ferroporphyrin-adducted cyclohexyl-fullerene – C_60_ “ball” (8, 9), is now proven of being safe and efficient nanodrug capable to correct some oxygen independent substrate phosphorylation disorders and also to modulate the “chaotically fluctuating” ATP / GTP production processes in aggressively proliferating mammalian cells (10, 11). This novel nanodrug, therefore, possesses some anti-cancer activity potential (12–14).

So PMC16 could be treated as a subject for the non-Markovian pharmacokinetics model (Figures 1, 2). This might be further developed towards an extrapolation of animal data to predict pharmacokinetic parameters by allometric scaling which is an often-used tool in drug development with multiple approaches available at variable success rates. In the most frequently used approach, pharmacokinetic parameters between different species are related via body weight using a power function:

(d) $$P=a\;•\;W^{b}$$

where *P* is the pharmacokinetic parameter scaled, *W* is the body weight in kilograms, *a* is the allometric coefficient, and b is the allometric exponent. *a* and *b* are specific constants for each parameter of a compound. General tendencies for the allometric exponent we estimated for PMC16 are 0.75 for rate constants (i.e. clearance, elimination rate constant), 1 for volumes of distribution, and 0.25 for half-lives (see *Results & Discussion*, Fig. 6).

For most traditional, small-molecule drugs, allometric scaling is often imprecise, especially if hepatic metabolism is a major elimination path way and/or if there are interspecies differences in metabolism (13). However, all hardly metabolized xenobiotics with molecular mass values higher than 2.0 kDa and, particularly, the metabolically stable carbon nanoparticles and their water-soluble derivatives were found to be perfectly suitable for allometric scaling “on the edge” between pre-clinical and clinical trial steps (9, 11, 12, 14–16).

## METHODS

For the *in vivo* administration, whatever animal (mouse, rat, rabbit, dog, goat) employed, a single intravenous injection of PMC16 dissolved in sterile physiological solution has been made as described in (9, 11). All animals were kept on a standard vitamins enriched diet and were starving for 24 hrs prior to experiment.

To estimate all major conventional pharmacokinetics patterns, a routine procedure specified in (11, 12) has been employed. In brief, the PMC16 identification/quantification in any biomaterial tested (urine, blood cells and plasma, tissue homogenates) was carried out using the lyophilized CS_2_/acetone-extracts treated by an original Trident-SLC4 / HPLC - MALDI chromato-mass-spectrometry technique in the Varian SQ400 LC-MS Analyser (10, 11).

The reagents used, all of Analytical grade, were purchased from the Bio-Rad Corp. Moscow Division, Moscow, Russia.

A non-parametric statistical treatment (for “n” equal to 6 or lower) was performed using the Sigma Biostat A6 software package to evaluate the significance of experiment/control differences. A Penmann-Dalbreaux approximation technique has then been employed once the standard error of the mean (SEM) values were found to be not higher than 6.5% of the mean (0.020–0.065 M limit range) whatever pair compared. To manage this, the HP LabRun-06 software operating graph-design algorithm has been processed in the HP6100-J2A Analytical Unit to reach the perfection for all graphics build.

## RESULTS AND DISCUSSION

**Figure 4:**
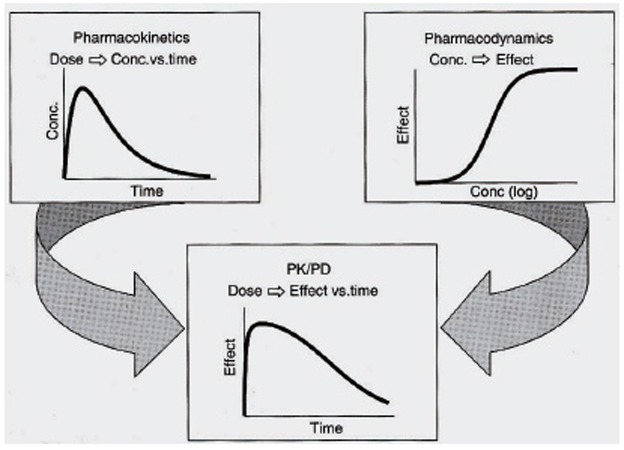
Pharmacokinetics/Pharmacodynamics (PK/PD) Combined analysis general scheme.

Pharmacokinetics / pharmacodynamics simultaneous (PK/PD) modeling does not only allow for a continuous description of the time course of effect as a function of the dosing regimen and comprehensive summary of available data but also enables testing of competing hypotheses regarding processes altered by the drug, allows to make predictions of drug effects under new conditions, and facilitates the estimation of inaccessible system variables (Figure 4).

The application of PK/PD modeling is beneficial in all phases of preclinical and clinical drug development, with a focus on dosage optimization and identification of covariates that are causal for intra- and interindividual differences in drug response and/or toxicity. It has recently further been endorsed by the publication of the Exposure-Response Guidance document by the U.S. Food and Drug Administration (14). Mechanism-based PK/PD modeling appreciating the physiological events involved in the elaboration of the observed effect has been promoted as superior modeling approach compared to empirical modeling, especially because it does not only describe observations but also offers some insight into the underlying biological processes involved and thus provides flexibility in extrapolating the model to other clinical situations (15).

While drug concentrations are usually analytically quantified in plasma, serum, or blood, the magnitude of the observed response is determined by the concentration of the drug at its effect site, the site of action in the target tissue. The relationship between the drug concentration in plasma and at the effect site may either be constant or undergo time-dependent changes. If equilibrium between both concentrations is rapidly achieved or the site of action is within plasma, serum or blood, there is practically a constant relationship between both concentrations with no temporal delay between plasma and effect site. In this case, measured concentrations can directly serve as input for a pharmacodynamic model. The most frequently used direct link pharmacodynamic model is the sigmoid E_max_-Model:

(e) $$\text{E}=\;\frac{E_{\text{max}}\cdot C^{n}}{{\text{EC}}_{50}^{n}+C^{n}}$$

**Figure 5:**
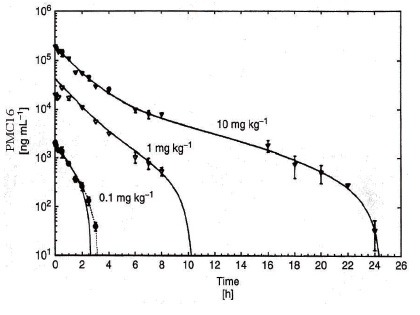
Nonlinear pharmacokinetics of pmc16, presented as measured and modeled plasma concentration - time curves (Mean ± SE) after a single intravenous injection of 0.1 mg kg^−1^ (*n* = 5), 1.0 mg kg^−1^ (*n* = 3), and 10 mg kg^−1^ (*n* = 8) in rats.

With *E_max_* as minimum achievable effect, *C* as drug concentration at the effect site, and *EC_50_* the concentration of the drug that produces half of the maximum effect. The Hill-coefficient *n* is a shape factor that allows for an improved fit of the relationship to the observed data. Thus, a direct link model directly connects measured concentration to the observed effect without any temporal delay. As seen from Figure 5, this was observed indeed in PMC16 pharmacokinetics experiments while the dose-interval regime parameters were in fact chosen for a drug-lymphocyte discrimination task pre-solved due to a non-Markovian algorithm employed earlier (Figures 1, 2).

**Figure 6:**
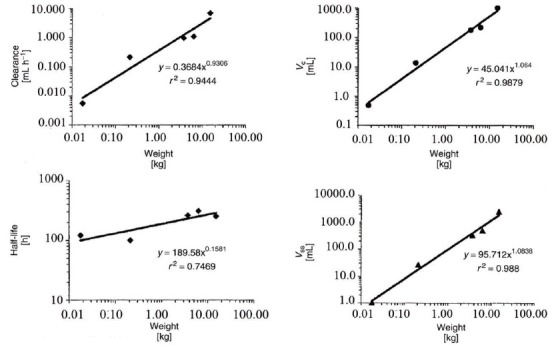
Allometric plots of the pharmacokinetic parameters: clearance, volume of the central compartment (*v_c_*), volume of distribution at steady state (*v_ss_*), and elimination half-life of pmc16. Each data point within the plot represents an averaged value of the pharmacokinetic parameter with increasing weight from mouse, rat, rabbit (3.5 kg), dog (6.3 kg), and goat, respectively. The solid line is the best fit with a power function to relate pharmacokinetic parameters to body weight.

All allometric plots obtained for the key PMC16 pharmacokinetics parameters (Figure 6) reveals the regularity surely positive for a clinical trial planning to come (16, 17).

A two-compartment model proposed (PMC16) is fitted to the following non-Markovian compatible pharmacokinetics data with both inter-individual and inter-occassional random effects on CL, V, Q, and V2 corrected to a error best described the pattern of residual error (17–19).

Inter-individual variability with covariate model:

A. Pharmacokinetic model
  (f) $$\text{C}=\frac{D}{V}\times [\frac{\left(\alpha -k_{21}\right)}{\alpha -\beta }\text{exp}-(\alpha \times t)+\frac{\left(k_{21}-\beta \right)}{\left(\alpha -\beta \right)}\text{exp}-(\beta \times t)]$$
B. Covariate model
  (g) $${\text{CL}}_{\text{j}}=[\theta_{3}\times {\text{OCC}}_{1}+\theta_{4}(\text{WT}-75)]\exp(\eta_{\text{CLj}})$$
  (h) $$V_{\text{cj}}=[\theta_{1}-(\text{GFR}-80)\times \theta_{2}]\times \text{exp}(\eta_{\text{vj}})$$
  (i) $${\text{k}}_{12\text{j}}=(\theta_{5})\times \text{exp}(\eta_{k12j})$$
C. Population pharmacokinetic model
  (J) $$Ci_{j}=\frac{D_{iv}}{\left[\theta_{1}\times {\text{OCC}}_{1}+\theta_{2}\times (\text{WT}-75)]\times \text{exp}(\eta_{v\;j}\right)}\left\{\begin{array}{l} \frac{k_{21}-\frac{CL_{j}}{V_{c\;j}}}{\beta -\frac{CL_{j}}{V_{c\;j}}}\times \text{exp}-(\frac{CL_{j}}{V_{c\;j}}\;t)\\ +\frac{k_{21}-k_{12}}{\frac{CL_{j}}{V_{c\;j}}-\beta }\times \text{exp}-({\text{k}}_{12}t) \end{array}\right\}\times \text{exp}(\varepsilon_{ij})$$
D. Population parameters
*θ*_1_ = 15.5
*θ*_2_ = 0.229

Thus, this peculiar segment of our present study is all about to get the pre-clinical trial (animal tests) derived information on the drug blood plasma level in humans which would be, in turn, minimally enough to provide an optimal drug-cell targeting distribution we may expect (predict) following our non-Markovian path in a chemotherapy strategy developments.

So the simulation approach we proposed would improve the efficacy of some new anti-cancer drugs pharmacokinetics/pharmacodynamics study at the edge of pre-clinical and clinical trials. This approach provides an opportunity to save time and resources owing to a simple prediction-power model that reveals the drug distribution among the targeted compartments with different turnover rates.
